# Understanding and Regulating C–N Coupling Pathways for Electrocatalytic Urea Synthesis From CO_2_ and NO_3_^−^/NO_2_^−^

**DOI:** 10.1002/advs.77499

**Published:** 2026-09-06

**Authors:** Xingbao Chen, Zhuo Li, Lebin Cai, Paul Kilmartin, Bao Yu Xia, Ziyun Wang

**Affiliations:** ^1^ School of Chemical Sciences University of Auckland Auckland New Zealand; ^2^ School of Chemistry and Chemical Engineering Huazhong University of Science and Technology Wuhan China

**Keywords:** C–N coupling mechanism, Intermediate regulation, urea electrosynthesis

## Abstract

Electrocatalytic co‐reduction of CO_2_ and NO_3_^−^/NO_2_^−^ to synthesize urea is a promising alternative to the traditional urea production process. However, the complexity of the reaction pathway hinders clear identification of C–N coupling mechanisms, thereby limiting the ability to guide catalyst design. Therefore, to design catalysts with improved performance, it is necessary to deepen the understanding of the urea synthesis process, particularly the C–N coupling step. This review summarises and discusses the current mechanistic insights into C–N coupling. Several representative C–N coupling pathways reported to date are reviewed and assessed in detail. Intermediate‐regulation strategies on both the carbon and nitrogen sides for promoting C–N coupling are discussed. In addition, commonly employed characterization techniques for elucidating the reaction mechanism are reviewed. Finally, this review provides perspectives on further exploring C–N coupling mechanisms.

## Graphical abstract

Understanding and regulating C–N coupling is central to electrocatalytic urea synthesis from CO_2_ and NO_3_^−^/NO_2_^−^. This review outlines representative coupling pathways, summarizes carbon‐ and nitrogen‐side intermediate regulation strategies, and highlights in situ/operando tools for mechanistic clarification and catalyst design.

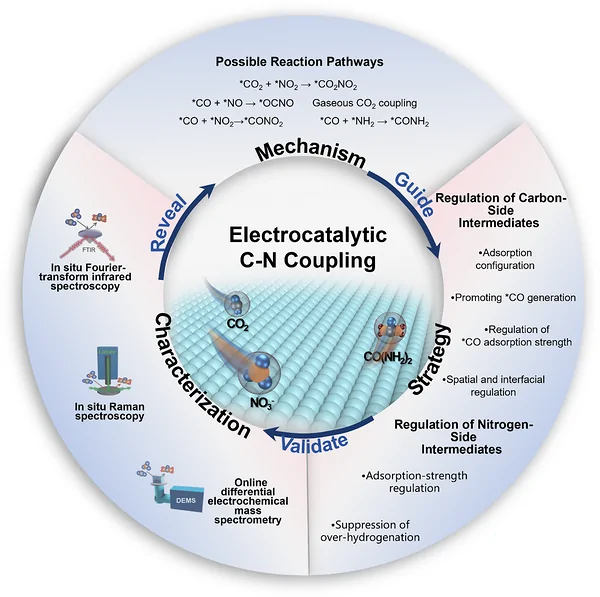

## 1 Introduction

Urea (CO(NH_2_)_2_) serves as a crucial artificial nitrogen fertilizer for global crop production [1] and is a key chemical feedstock in the synthesis of plastics, resins, and pharmaceuticals [2, 3]. Present industrial synthesis of urea mainly employs the Bosch‐Meiser method, in which NH_3_ and CO_2_ react at high temperature (150–200°C) and pressure (15–25 MPa) [4]. In addition, NH_3_, as one of the reactants, should be synthesized by the Haber‐Bosch method, which also requires harsh conditions, i.e., 400–600°C and 200–350 atm [5]. Therefore, the recent industrial two‐step process synthesis of urea consumes approximately 1%–2% of the global annual energy and generates a substantial amount of CO_2_ [6]. By contrast, electrochemical co‐reduction of CO_2_ and N‐containing (N_2_, NO, NO_2_^−^, and NO_3_^−^) sources to urea offers an environmentally friendly solution to reduce energy consumption [7, 8, 9, 10]. Compared to N_2_, which has a highly stable triple bond (N≡N, ∼941 kJ mol^−1^) [11], the bond energies of the N═O bonds in NO_2_^−^ and NO_3_^−^ are significantly lower. Moreover, these species are abundant in industrial wastewater, and their infiltration into groundwater causes various diseases, such as methemoglobinemia [12]. Therefore, NO_2_^−^ and NO_3_^−^ are regarded as ideal nitrogen sources for co‐reduction with CO_2_ in electrocatalytic urea synthesis [13, 14, 15].

Benefiting from the inherent advantages of electrochemical co‐reduction of CO_2_ and NO_3_^−^/NO_2_^−^ to urea, this field has attracted growing attention [9, 16, 17, 18]. Many strategies have been applied in catalyst design, for example, oxides [19], alloying [20], heteroatom doping [10, 21], vacancy engineering [22], facet regulation [23], and single‐atom engineering [24]. However, the understanding of the C–N coupling mechanism remains ambiguous owing to the intricate urea synthetic pathway, which hinders the rational design of catalysts with satisfactory performance [25]. The urea synthetic pathway involves 16 proton‐coupled electron transfer steps and two C–N coupling steps. For carbon species, gaseous CO_2_ and the other three possible intermediates may participate in the coupling process: *CO_2_, *COOH, and *CO. Furthermore, nitrogen species are more diverse and complex, with up to 13 intermediates theoretically existing between *NO_3_ and *NH_2_, all of which could potentially serve as coupling partners during C–N bond formation: i)*NO_3_ → *NO_2_OH → *NO_2_ → *NOOH → *NO → *NOH → *N → *NH → *NH_2_; ii) *NO_3_ → *NO_2_OH* → *NO_2_ → *NOOH → *NO → *HNO → *NHOH → *NH_2_OH → *NH_2_; iii) *NO_3_ → *NO_2_OH → *NO_2_ → *NOOH → *NO → *HNO → *ONH_2_ → *NH_2_OH → *NH_2_ [26]. Therefore, it is essential to understand which intermediates dominate the C–N coupling process and how they interact with specific catalytic surfaces.

In this review, we summarize recent developments within mechanistic studies in the electrocatalytic co‐reduction of CO_2_ and NO_3_^−^/NO_2_^−^ to urea, focusing on three major aspects: (i) mechanistic insights into C–N coupling derived from theoretical and experimental studies; (ii) intermediate regulation strategies toward efficient C–N coupling; and (iii) advances in in situ characterization techniques that have enhanced our understanding of intermediate species and coupling mechanism. By systematically reviewing recent progress in these areas, we aim to provide a comprehensive understanding of the field and offer guidance for future research directions. Finally, the current challenges and perspectives on uncovering C–N coupling mechanisms are discussed.

## 2 Representative C–N Coupling Pathways in Electrocatalytic Urea Synthesis

**FIGURE 1:**
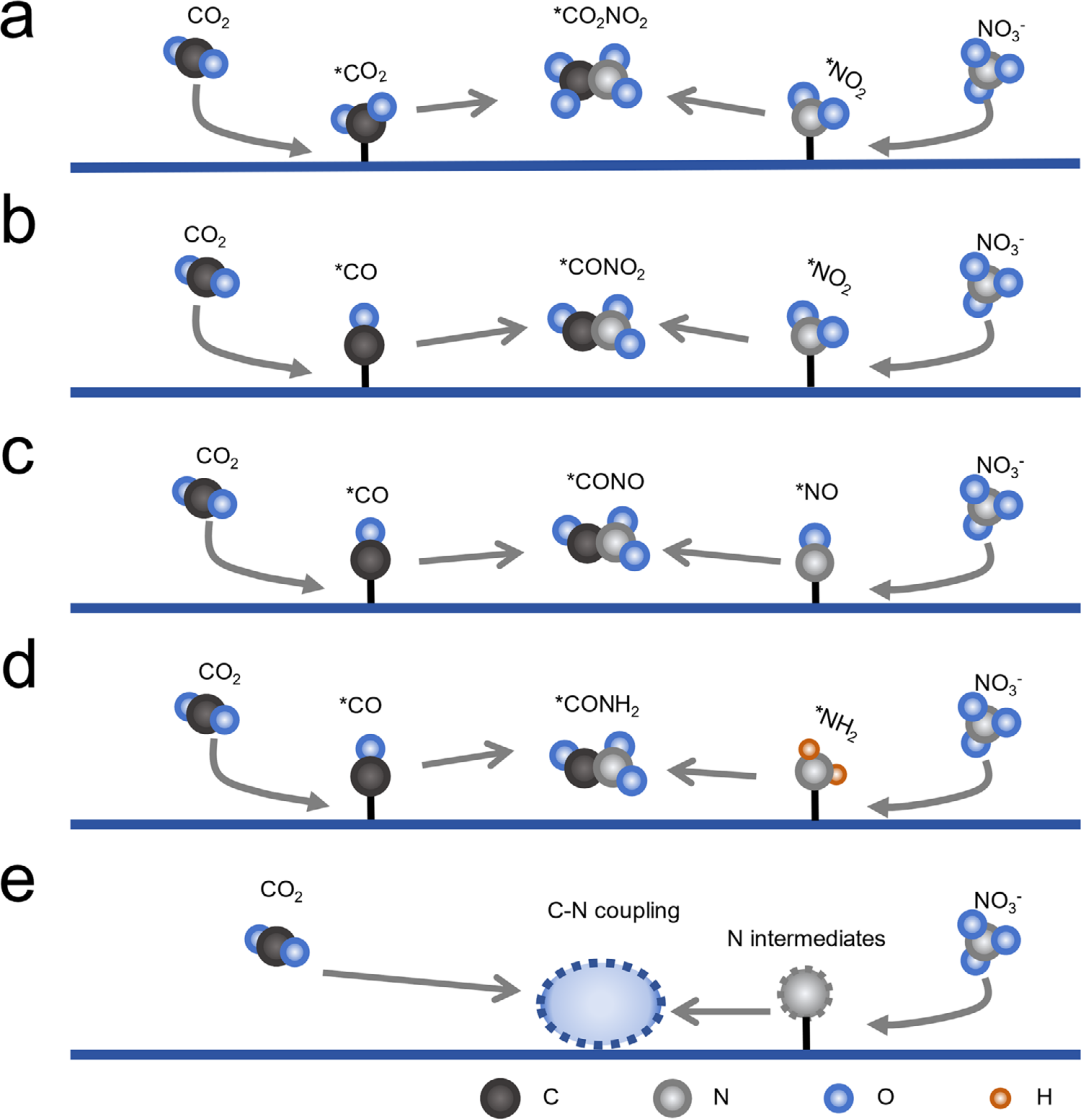
C–N coupling mechanisms of CO_2_ and NO_3_^−^ co‐reduction to urea. (a) *CO_2_ + *NO_2_ → *CO_2_NO_2_, (b) *CO + *NO_2_ → *CONO_2_, (c) *CO + *NO → *OCNO, (d) *CO + *NH_2_ → *CONH_2_, (e) direct coupling with gaseous CO_2_.

Current studies indicate that the first C–N coupling step is critical for CO_2_ and NO_3_^−^/NO_2_^−^ co‐reduction to urea [27, 28, 29]. Based on recent studies, the proposed mechanisms of first C–N coupling step can be broadly categorized into five representative types: (i) *CO_2_ + *NO_2_ → *CO_2_NO_2_; (ii) *CO + *NO_2_ → *CONO_2_; (iii) *CO + *NO → *OCNO; (iv) *CO + *NH_2_ → *CONH_2_; (v) direct coupling with gaseous CO_2_ (Figure 1 and Table 1). The following sections elaborate on each proposed C–N coupling pathway, highlighting the adsorption behaviors on specific catalyst types, key coupling intermediates, and supporting experimental or theoretical evidence.

**TABLE 1:** Recent catalysts for NO_3_^−^/NO_2_^−^ and CO_2_ reduction to urea.

C–N coupling	C and N source	Catalyst	FE (%)	Yield rate	References
*CO_2_NO_2_	CO_2_ + NO_3_^−^	In(OH)_3_‐S	53.4	533.1 µg h^−1^ mg_cat._^−1^	[31]
	CO_2_ + NO_3_^−^	MoO_x_/C	27.7	1431.5 µg h^−1^ mg_cat._^−1^	[30]
	CO_2_ + NO_3_^−^	V_O_‐S‐IO‐6	60.6	910.4 µg h^−1^ mg_cat._^−1^	[53]
	CO_2_ + NO_3_^−^	FeN_4_/B_2_CuN_2_@NC	71.9	2072.5 µg h^−1^ mg_cat._^−1^	[54]
	CO_2_ + NO_3_^−^	PdAuCuIrCo	22.57	52.43 mmol h^−1^ g^−1^	[55]
	CO_2_ + NO_3_^−^	3D Cu/Zn	75	16 µmol h^−1^ cm^−2^	[7]
	CO_2_ + NO_3_^−^	U‐Zn	31.8	39.3 mmol h^−1^ g^−1^	[32]
*CONO_2_	CO_2_ + NO_3_^−^	CuWO_4_	70.1	98.5 µg h^−1^ mg_cat._^−1^	[33]
	CO_2_ + NO_3_^−^	Cu‐Bi_0.1_	89.4	586.87 µg h^−1^ mg_cat._^−1^	[56]
	CO_2_ + NO_3_^−^	IL@Cu‐ZIF‐8	55.3	140 µmol h^−1^ cm^−2^	[57]
	CO_2_ + NO_3_^−^	CuSiO_X_	79.01	1606.1 µg h^−1^ mg_cat._^−1^	[34]
*OCNO	CO_2_ + NO_3_^−^	Cu_1_–CeO_2_	—	52.84 mmol h^−1^ g_cat._^−1^	[35]
	CO_2_ + NO_3_^−^	Cu‐PMOF	25.5	25.5 mmol h^−1^ mg_cat._^−1^	[36]
	CO_2_ + NO_3_^−^	O–Bi_M_/CuO_X_	23.5	2180.3 µg h^−1^ mg_cat._^−1^	[58]
	CO_2_ + NO_3_^−^	V_O_‐CeO_2_‐750	—	943.6 mg h^−1^ g^−1^	[38]
	CO_2_ + NO_3_^−^	Cu_1_Au_8_@CeO_2_	45.2	813.6 µg h^−1^ mg_cat._^−1^	[59]
	CO_2_ + NO_3_^−^	N‐C‐1000	—	610.6 mg h^−1^ g^−1^	[37]
	CO_2_ + NO_3_^−^	Zn–Fe_2_O_3_/O_V_	62.4	7.48 mg h^−1^ mg_cat._^−1^	[60]
*CONH_2_	CO_2_ + NO_3_^−^	Ni@CeO_2‐X_	70.1	2175.47 µg h^−1^ mg^−1^	[39]
	CO_2_ + NO_3_^−^	SrCo_0.39_Ru_0.61_O_3−δ_	34.1	1522 µg h^−1^ mg^−1^	[61]
	CO_2_ + NO_3_^−^	CuZn	40	304.8 mmol h^−1^ g_cat._^−1^	[62]
	CO_2_ + NO_3_^−^	Fe(a)@C‐Fe_3_O_4_/CNTs	16.5	1341.3 µg h^−1^ mg_cat._^−1^	[49]
	CO_2_ + NO_3_^−^	Pd_4_Cu_1_/FeNi(OH)_2_	66.4	436.9 mmol h^−1^ g_cat._^−1^	[63]
	CO_2_ + NO_3_^−^	Cu_AC‐_Cu_SA_@NC	—	42.4 mmol h^−1^ g_cat._^−1^	[64]
	CO_2_ + NO_3_^−^	Cu‐N_4−x_‐C_x_	28	—	[24]
	CO_2_ + NO_3_^−^	Mo‐PCN‐222(Co)	33.90	844.11 mg h^−1^ g^−1^	[65]
	CO_2_ + NO_3_^−^	Cu‐GS‐800	28	4.3 nmol s^−1^ cm^−2^	[24]
	CO_2_ + NO_3_^−^	FeP_0.9_S_2.9‐x_	15.4	1160.9 µg h^−1^ mg_cat._^−1^	[66]
	CO_2_ + NO_3_^−^	Cu_1.0_/ZnO_0.5_	37.4	3.2 µmol h^−1^ cm^−2^	[67]
	CO_2_ + NO_3_^−^	Fe_acs_	39.8	3643.65 mM h^−1^ g_Fe_^−1^	[68]
	CO_2_ + NO_3_^−^	Ga/Y‐CNP	22.1	41.9 mmol h^−1^ g^−1^	[69]
	CO_2_ + NO_2_^−^	Co–NiOx@GDY	64.3	913.2 µg h^−1^ mg_cat_^−1^	[41]
	CO_2_ + NO_2_^−^	Te–Pd NCs/C	12.2	—	[10]
	CO_2_ + NO_2_^−^	AuCu SANFs	24.7	3,889.6 µg h^−1^ mg_cat._^−1^	[40]
Gaseous CO_2_ coupling	CO_2_ + NO_3_^−^	B‐Cu	80.1	101.2 µmol h^−1^ cm^−2^	[21]
	CO_2_ + NO_3_^−^	MIL‐125‐NH_2_‐derived TiO_2_	48.88	43.37 mmol h^−1^ g^−1^	[4]

*Note*: Urea yield rates are reported in the original units from the cited literature.

### 2.1 *CO_2_ + *NO_2_ → *CO_2_NO_2_

**FIGURE 2:**
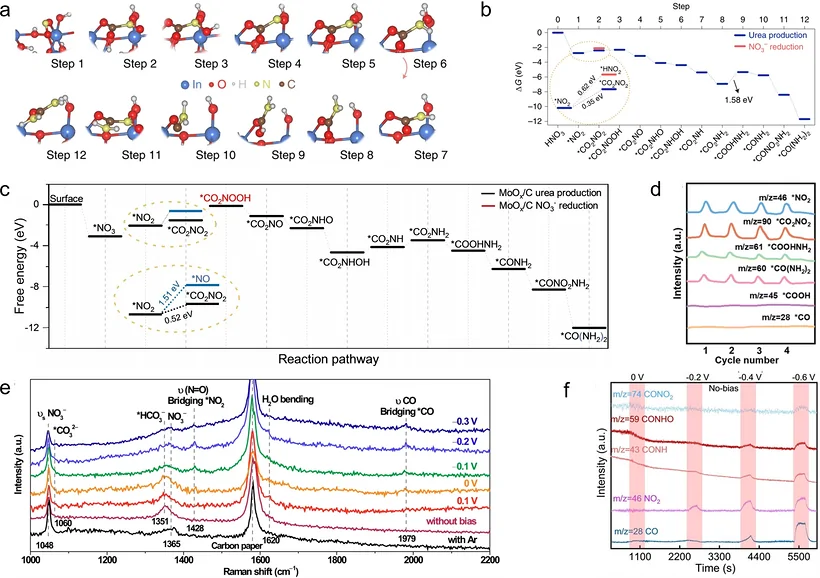
(a) Corresponding atomic configurations for each step of urea production on In(OH)_3_. (Adapted with permission from Ref. [31], copyright 2021 Springer Nature). (b) The proposed mechanism of urea synthesis through DFT on In(OH)_3_‐S. (Adapted with permission from Ref. [31], copyright 2021 Springer Nature). (c) Comparison of Gibbs free energy changes associated with urea formation on MoO_x_/C and MoO_3_. (Adapted with permission from Ref. [30], copyright 2023 Wiley‐VCH). (d) Online DEMS spectra of U‐Zn. (Adapted with permission from [32], copyright 2025, Royal Society of Chemistry). (e) In situ Raman spectra of CuWO_4_ in 0.1 M KNO_3_ under CO_2_ flow at various potentials and under Ar at open circuit. (Adapted with permission from Ref. [33], copyright 2023 Springer Nature). (f) Online DEMS spectra of CuSiO_X_ in 0.1 M KNO_3_ and 0.1 M KHCO_3_ under CO_2_ at various potentials. (Adapted with permission from Ref. [34], copyright 2024 Wiley‐VCH).

Formation of the *CO_2_NO_2_ intermediate occurs at an early stage of the co‐reduction, typically after the initial two‐electron reduction of nitrate to surface‐bound *NO_2_. Subsequently, adsorbed *CO_2_ on the catalyst surface couples with *NO_2_ to yield *CO_2_NO_2_. Since the coupling intermediates require relatively fewer hydrogenation steps, the mechanism tends to reduce by‐product formation and deliver higher urea selectivity at comparatively lower overpotentials [30]. Through operando synchrotron‐radiation Fourier transform infrared (SR‐FTIR) spectroscopy measurements and density functional theory (DFT) calculations, Lv et al. initially reported that the surface‐bound *CO_2_ can directly couple with absorbed *NO_2_ on In(OH)_3_‐S catalyst, particularly on the (100) facet [31]. As a first step, *NO_2_ adsorbs on coordinatively unsaturated indium sites, where electron transfer to the adsorbate enhances its reactivity toward C–N coupling. Simultaneously, electron transfer from the indium sites to *CO_2_ facilitates the C–N coupling step between *CO_2_ and *NO_2_ (Figure 2a). Notably, DFT calculations show that the energy barrier for *NO_2_ hydrogenation to *HNO_2_ (0.62 eV) is higher than that for coupling with *CO_2_ (0.35 eV), demonstrating that the C–N coupling step is kinetically favored (Figure 2b). As a result, In(OH)_3_‐S exhibits outstanding electrocatalytic performance, achieving a Faradaic efficiency of 53.4% for urea, as well as nitrogen selectivity of 82.9% and carbon selectivity approaching 100%.

Similarly, Sun et al., reported that *CO_2_ directly couples with *NO_2_ to form *CO_2_NO_2_ on MoO_x_/C catalysts, thereby initiating urea formation [30]. Mechanistic insights into the urea formation pathway were unveiled by DFT calculations through identifying the most energetically favorable reaction route. In detail, the process of urea formation begins with the reduction of NO_3_^−^ to *NO_2_. Subsequently, *NO_2_ faces two competitive reaction pathways: coupling with *CO_2_ or hydrogenation to form *NO. Notably, *NO_2_ preferentially undergoes C–N coupling with *CO_2_ to form a *CO_2_NO_2_ intermediate, as this step requires a substantially lower energy barrier compared with its further reduction to *NO (Figure 2c). The lower C–N coupling energy barrier leads to a Faradaic efficiency of NO_3_^−^ reduction to NH_3_ as high as 98.14% in the absence of CO_2_. However, when CO_2_ is introduced, the Faradaic efficiency of NH_3_ decreases to 14.59%, and achieves a urea Faradaic efficiency of 27.7%. Wang et al., further demonstrated the *NO_2_ and *CO_2_ coupling mechanism on uncoordinated Zn nanosheets through online differential electrochemical mass spectrometry (DEMS) [32]. The signals of *NO_2_, *CO_2_NO_2_, *COOHNH_2_, and *CO(NH_2_)_2_ were observed, but the signals of *COOH and *CO were absent (Figure 2d). The result suggests that the coupling step occurs between *CO_2_ and *NO_2_.

In summary, the C–N coupling process can directly occur between adsorbed *CO_2_ and *NO_2_, without the need for a deep hydrogenation process with these intermediates. In this mechanism, the C–N bond formation at the early stage of nitrate reduction allows *NO_2_ to participate in the coupling reaction before it further converts into more deeply reduced intermediates such as *NO or *NH_x_. This coupling mode effectively inhibits the competition of the continuous hydrogenation pathway in terms of kinetics, thereby reducing the tendency of the formation of ammonia and other by‐products, and facilitating the selection of the reaction to form urea. Therefore, the direct coupling of *CO_2_ and *NO_2_ provides an available reaction pathway for urea production.

### 2.2 *CO + *NO_2_→*CONO_2_

*CO and *NO_2_ as coupling intermediates were proposed by Zhao et al. CO_2_ undergoes two hydrogenation steps to yield *CO, which then acts as the carbon‐containing intermediate [33]. Simultaneously, NO_3_^−^ is reduced to *NO_2_ through proton‐coupled electron transfer steps. These two surface‐adsorbed species, *CO and *NO_2_, then couple to initiate the C─N bond formation process. In situ Raman results revealed the peaks of *CO and *NO_2_ at 1979 cm^−1^ and 1428 cm^−1^, respectively. Notably, the peak intensity reached its maximum at −0.2 V vs. RHE, which coincided with the potential at which the highest urea yield and FE_urea_ were observed (Figure 2e). This correlation provided evidence that *CO and *NO_2_ are likely the key intermediates involved in the C–N coupling process. Additionally, a series of carbon‐ and nitrogen‐containing reactants was tested to validate the proposed mechanism. The results revealed that when any downstream intermediates beyond *CO and *NO_2_ were introduced into the reaction system, urea formation was not detected, further supporting the role of *CO and *NO_2_ as key coupling intermediates on CuWO_4_. Qiu et al. also proposed the *CO + *NO_2_ coupling mechanism on a CuSiO_X_ catalyst, which was validated through in situ attenuated total reflection Fourier transform infrared spectroscopy (ATR‐FTIR) spectroscopy, DEMS, and DFT calculations [34]. As shown in Figure 2f, the intermediate of *CONH (m/z = 43), *CONHO (m/z = 59), and *CONO_2_ (m/z = 74) were observed. Additionally, the *CONO_2_ intermediate was detected by in situ ATR‐FTIR spectroscopy, with a characteristic adsorption peak observed at 1695 cm^−1^. Among all experimentally detected intermediates, *CONO_2_ appears at the earliest stage of the hydrogenation pathway, suggesting its potential role as the initial product of C–N coupling.

Generally, nitrate reduction (NO_3_RR) or nitrite reduction (NO_2_RR) initiates at more positive potentials than CO_2_ reduction (CO_2_RR), making it difficult to simultaneously sustain high surface coverages of *NO_2_ and *CO on most catalysts. To exploit the *NO_2_ and *CO coupling pathway, catalysts must be rationally engineered to (i) lower the overpotential for converting CO_2_ to *CO and/or (ii) raise the activation barrier for the further reduction of *NO_2_, thereby sustaining concurrent high coverages of both intermediates.

### 2.3 *CO + *NO → *OCNO

**FIGURE 3:**
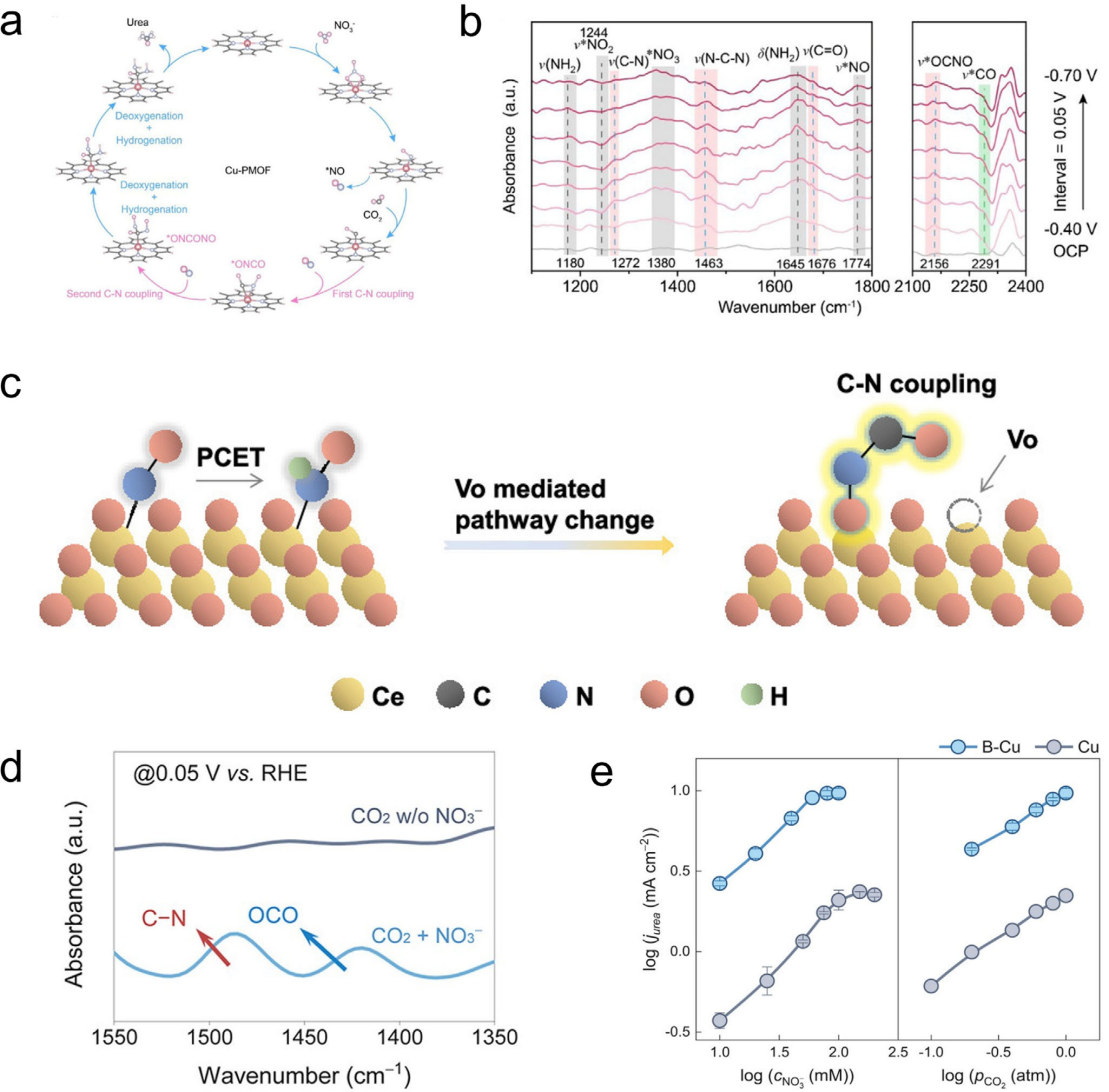
(a) Schematic illustration of the urea synthetic routes for Cu‐PMOF. (Adapted with permission from Ref. [36], copyright 2025 Wiley‐VCH). (b) In situ ATR‐SEIRAS spectra at various applied potentials for Cu‐PMOF in 0.05 M NO_3_^−^. (Adapted with permission from Ref. [36], copyright 2025 Wiley‐VCH). (c) Schematic diagram of *CO and *NO coupling mechanism on V_O_‐CeO_2_. (Adapted with permission from Ref. [38], copyright 2022 American Chemical Society). (d) In situ FTIR spectra collected in CO_2_‐saturated electrolyte with and without NO_3_^−^ at 0.05 V vs. RHE. (Adapted with permission from Ref. [21], copyright 2025 American Chemical Society). (e) c_NO3‐_ and p_CO2_‐dependent urea formation rate on B–Cu and Cu catalysts at 0.05 V vs. RHE. (Adapted with permission from Ref. [21], copyright 2025 American Chemical Society).

Another proposed C–N coupling mechanism involves the reaction between *CO and *NO to form *OCNO, which has been identified as a key intermediate in several recent studies [35, 36, 37]. Tan et al. introduced the coupling mechanism on Cu‐PMOF (Figure 3a) and proved that via in situ Attenuated Total Reflection Surface‐Enhanced Infrared Absorption Spectroscopy (ATR‐SEIRAS) [36]. The peaks at 1774, 2291, and 2156 cm^−1^ were attributed to the stretching vibrations of *NO, *CO, and *OCNO, respectively, demonstrating the *CO + *NO → *OCNO pathway (Figure 3b). Online DEMS was employed to identify the key intermediates involved in the urea synthesis process. Cu‐PMOF exhibited signals of at m/z = 30, 58, and 88, which correspond to *NO, *ONCO, and *ONCONO intermediates, respectively. They suggested that *NO and *CO were absorbed on the same Cu site and formed a *OCNO intermediate with no apparent structural changes. Therefore, this coupling process is kinetically favorable, as indicated by a negative Gibbs free energy change (ΔG < 0). The hydrogenation of *CO_2_ to *CO requires the highest energy barrier in the overall reaction (ΔG = 0.21 eV), so this step is identified as the rate‐determining step. Wei et al. uncovered a distinct *CO + *NO coupling pathway on V_O_‐enriched CeO_2_, in which *NO inserts into oxygen vacancies and couples directly with *CO to form *OCNO rather than conventional protonation routes (Figure 3c) [38]. The kinetic barriers for protonation and coupling steps were calculated through DFT, demonstrating that the energy barrier of *CO and *NO coupling reaction on V_O_‐enriched CeO_2_ only needs 0.27 eV, lower than *NO protonation (1.75 eV) and *CO coupling with *NH (0.85 eV)or *NH_2_ (0.85 eV). Meanwhile, Wang and his colleagues used operando SR‐FTIR to observe the coupling of *NO and *CO to form the key *OCNO intermediate, indicating that the first C–N bond could occur on the metal‐free catalyst (N‐C‐1000) [37]. These studies provide evidence that the *CO + *NO coupling pathway, involving the formation of *OCNO as a key intermediate, can occur on a variety of catalyst surfaces, including both metal‐based and metal‐free systems.

### 2.4 *CO + *NH_2_ → *CONH_2_

The coupling pathway between *CO and *NH_2_ is another frequently proposed reaction route. This mechanism suggests that the C–N bond formation occurs at a relatively late stage of the overall reaction. Specifically, CO_2_ undergoes two proton‐coupled electron transfer steps to form *CO, while NO_3_^−^ is reduced through seven electron transfer steps to yield *NH_2_. Subsequently, *CO couples with *NH_2_ to form the *CONH_2_ intermediate, which then undergoes a second coupling step with another *NH_2_ species to finally generate urea. Liang et al. confirmed the *CONH_2_ mechanism on Ni‐CeO_2_‐X via in situ ATR‐FTIR substitution experiments with various C‐ and N‐sources [39]. They utilized CO_2_, CO, HCHO, and CH_3_OH as carbon sources and NO_3_^−^ as the nitrogen source. Urea formation was only observed when CO_2_ or CO was employed. These results demonstrate that *CO can act as a reactive intermediate in the C–N coupling process. When CO_2_ was used as the carbon source and KNO_2_, NH_2_OH, HCONH_2_, NH_3_, and NH_4_Cl were separately used as nitrogen sources, urea was detected only in systems with KNO_2_, NH_2_OH, or HCONH_2_. The detection of urea when using HCONH_2_ as the nitrogen source suggests that *CONH_2_ may serve as a key intermediate in urea synthesis. This intermediate is likely formed through the coupling of *CO and *NH_2_, indicating that *NH_2_ is a crucial N‐containing species involved in the C–N coupling process. Moreover, numerous studies on NO_2_^−^ reduction systems have identified *CONH_2_ as the initial C–N coupling intermediate in urea synthesis [10, 22, 40, 41]. For example, Feng et al. reported the *NH_2_ and *CO coupling mechanism in NO_2_^−^ and CO_2_ co‐reduction system on Te‐doped Pd nanocrystals [10].

The coupling mechanism of *CO and *NH_2_ relies on the deep reduction process of CO_2_ and NO_3_^−^/NO_2_^−^. Therefore, under this mechanism, the C–N coupling usually occurs in a more negative reaction potential range. In this mechanism, *CO and *NH_2_ represent the last active intermediates that the carbon‐containing and nitrogen‐containing intermediates, respectively, which can participate in coupling for generating urea. Further reduction of either intermediate diverts the reaction pathway toward competing side products such as CO and NH_3_. Therefore, in the *CO and *NH_2_ coupling mechanism, promoting the effective generation of both while inhibiting their excessive reduction is critical for achieving high‐selectivity urea synthesis.

### 2.5 Gaseous CO_2_ Coupling Mechanism

Aqueous amine solutions have been widely employed in industrial CO_2_ capture, particularly for treating low‐concentration CO_2_ streams such as postcombustion flue gas (10%–15% CO_2_), owing to the strong affinity between CO_2_ molecules and amino functional groups [42, 43]. During the NO_3_^−^ and NO_2_^−^ reduction processes, a variety of surface‐adsorbed ammonia‐related intermediates are commonly generated. Therefore, the ammonia intermediates formed during the electrochemical reduction of NO_3_^−^ or NO_2_^−^ may serve as active sites for C–N coupling through chemisorptive interaction with CO_2_. Hu et al. proposed a coupling mechanism in which *NO_2_^−^ captures CO_2_ through the Eley–Rideal (ER) mechanism to facilitate C–N bond formation [21]. In situ FTIR was used to uncover the C–N coupling mechanism. At 0.05 V vs. RHE, no *OCO signal was detected when only CO_2_ was present, suggesting that CO_2_ alone could not be activated on Cu. By contrast, in the CO_2_ + NO_3_^−^ system, both the C─N bond signal associated with urea and the *OCO signal were observed, supporting that C–N coupling proceeds via direct participation of gaseous CO_2_ (Figure 3d). They further investigated the C–N coupling mode by adjusting the concentration of NO_3_^−^ and the partial pressure of CO_2_. When the concentration of NO_3_^−^ increased from 0.01 to 0.2 M, the rate of urea production initially rose significantly, but gradually plateaued at approximately 0.06 M, indicating that the surface coverage of *NO_2_ had reached saturation. In contrast, as the partial pressure of CO_2_ increased from 0.1 to 1 atm, the rate of urea production continued to increase without showing a saturation trend (Figure 3e). This kinetic behaviour indicates that CO_2_ does not participate in the reaction through surface adsorption of intermediates but directly couples with the adsorbed *NO_2_ in gaseous form, which is consistent with an ER mechanism. In further research, the same ER mechanism was also observed on Fe_2_O_3_ and Cd‐Fe_2_O_3_ [44]. These studies collectively suggest that various nitrogen‐containing intermediates can interact with CO_2_, enabling alternative pathways for C─N bond formation.

The gaseous CO_2_ coupling pathway offers a novel strategy to enhance urea selectivity. This route does not require prior activation of CO_2_ to *CO_2_, *COOH, or *CO, steps that typically demand more negative potentials. Employing this mechanism lowers the operating overpotential for urea electrosynthesis, thereby suppressing competing reactions. In addition, this mechanism provides an easy‐to‐handle and effective strategy to enhance the kinetic favorability of C–N coupling by increasing the local CO_2_ concentration at the catalyst surface.

### 2.6 Thermodynamic and Kinetic Analysis for Different C–N Coupling Pathways

As discussed above, different C–N coupling pathways are often manifested on different catalyst surfaces. This suggests that no single coupling pathway is universally favored. Instead, the dominant pathway depends on the thermodynamic and kinetic characteristics of the catalytic system. Furthermore, operating conditions, particularly the applied potential and electrolyte pH, could alter thermodynamic and kinetic characteristics. Therefore, it is important to consider the catalytic conditions in which these mechanisms are more likely to occur, as well as the fundamental thermodynamic and kinetic differences between pathways.

For the *CO_2_ + *NO_2_ → *CO_2_NO_2_ pathway, C–N coupling occurs before extensive reduction of the carbon and nitrogen intermediates, which tends to suppress the formation of by‐products. However, the feasibility of this pathway is still constrained by the behavior of both C‐ and N‐intermediates. From a thermodynamic perspective, the adsorption of CO_2_ to *CO_2_ is often considered the rate‐determining step for CO_2_RR [45, 46]. As a result, on active CO_2_RR catalysts, after the formation of *CO_2_, further hydrogenation to *COOH or *OCHO is favorable. This suggests that preventing the further hydrogenation of CO_2_, thereby enabling its participation in C–N coupling at an early stage of reduction, is a consideration for this mechanism. In the NO_3_RR process, the rate‐determining step varies across catalysts. The hydrogenation of *NO_2_ on catalysts is generally regarded as one of the rate‐determining steps during NO_3_RR [47, 48]. Therefore, from an energetic perspective, the relatively high activation energy of *NO_2_ hydrogenation hinders its further reduction, making the coupling with *CO_2_ more competitive. From a kinetic perspective, this can also lead to the accumulation of *NO_2_ on catalyst surfaces, facilitating C–N coupling with *CO_2_. In contrast, on those catalysts where the hydrogenation of *NO_2_ is not the rate‐determining step, the hydrogenation of *NO_2_ is relatively easier, which kinetically suppresses the *NO_2_ + *CO_2_ coupling pathway. Overall, the early coupling pathway of *CO_2_ + *NO_2_ relies on the stability of *NO_2_ and the hindered process of *CO_2_ hydrogenation. Therefore, this mechanism is more common in systems where the adsorption of *CO_2_ is weak, or CO_2_RR activity is limited, and the energy barrier for *NO_2_ hydrogenation is relatively high.

Different from the early coupling pathway, C–N coupling occurs at a later reduction stage, generally between *CO and *NO_2_ or *NO. From the energetic standpoint, C–N coupling between *CO and *NO_2_/*NO generally does not involve significant energy barriers. Therefore, this pathway has been proposed as feasible in many theoretical computational studies [27]. However, the actual occurrence is often limited by kinetic factors, namely whether C‐ and N‐intermediates can coexist within the same potential window. Specifically, the generation of *CO from CO_2_RR typically requires a more negative potential, while the hydrogenation of *NO_2_ and *NO usually occurs over a more positive potential range [33]. If the active potential ranges of these processes are mismatched, it becomes difficult to establish sufficient surface coverage of both *CO and *NO_2_/*NO simultaneously, which limits C–N coupling through this pathway. Therefore, these coupling pathways are more favorable on catalysts that combine a low overpotential for CO_2_ to CO conversion and relatively high energy barriers for *NO_2_/*NO hydrogenation.

The *NH_2_ + *CO coupling pathway is a frequently discussed coupling route because it typically exhibits a relatively low energy barrier [10, 49]. However, the hydrogenation of *NH_2_ to *NH_3_ often also proceeds with a relatively low energy barrier [50, 51]. Therefore, from a kinetic perspective, this pathway is mainly constrained by the stability of *NH_2_. When hydrogenation of *NH_2_ towards *NH_3_ occurs rapidly, it is difficult to maintain an adequate surface coverage of *NH_2_, thereby limiting its coupling with *CO. This coupling pathway can become dominant only when the hydrogenation of *NH_2_ is sufficiently suppressed, allowing *NH_2_ to remain on the surface and maintain sufficient coverage for C–N bond formation. Consequently, this mechanism is more likely to occur in catalytic systems that can slow the hydrogenation of *NH_2_.

Unlike the Langmuir‐Hinshelwood (LH) coupling pathways discussed above, which require the participation of two adsorbed intermediates, the ER mechanism allows gaseous CO_2_ to directly couple with surface nitrogen intermediates. Therefore, this pathway can occur in catalyst systems without CO_2_RR activity. Under this mechanism, the *CO_2_ adsorption step can be bypassed, thereby avoiding side reactions arising from the further reduction of carbon species [44]. Meanwhile, in a flow cell system, CO_2_ can maintain a relatively high local concentration at the interface, providing favorable kinetic conditions for direct coupling between gaseous CO_2_ and surface nitrogen species. However, the direct coupling of inactivated linear CO_2_ molecules with nitrogen species might require a higher reaction energy barrier. Compared to the coupling pathways involving *CO_2_, the thermodynamic feasibility of the ER process may be lower. Therefore, lowering the reaction barrier to gaseous CO_2_ coupling by regulating the catalytic interface or local environment might be a key challenge for promoting this C–N pathway.

Through an in‐depth analysis and summary of current C–N coupling mechanisms, it is evident that the C─N bond formation pathway largely depends on the adsorption behavior of key intermediates and their associated reaction energy barriers on the catalyst surface. Different catalyst surfaces support distinct C–N coupling routes, which significantly influence the selectivity and energy efficiency of urea formation. Therefore, before focusing on enhancing catalyst performance, it is essential to first identify and understand the feasible C–N coupling pathways specific to a given catalytic system. This strategic approach is a critical prerequisite for advancing research in CO_2_ and NO_3_^−^/NO_2_^−^ co‐reduction to urea.

## 3 Intermediate Regulation Strategies for Efficient C–N Coupling

Efficient electrocatalytic urea synthesis from CO_2_ and nitrate fundamentally relies on the formation of C–N bonds between carbon‐ and nitrogen‐derived intermediates at the electrode interface. Numerous catalyst design strategies have been reported to promote C–N coupling [7]. Currently, researchers mainly focus on enhancing the selectivity of electrochemical urea synthesis through refined material design. However, from a mechanistic perspective, regardless of how complex the catalyst structure is, the fundamental improvement in performance still stems from the regulation of the generation, stability, and transformation behavior of key intermediates during the reaction process. In other words, material design is not the goal; rather, it is through altering the interface electronic structure and adsorption properties to effectively guide the reaction pathways of intermediates, thereby ultimately influencing the C–N coupling efficiency and urea selectivity. Therefore, by concentrating the research strategy for enhancing urea selectivity on the regulation of the reaction behavior of key intermediates, it will help provide more mechanism‐oriented guidance for subsequent studies [52].

Accordingly, this chapter examines regulation strategies for C–N coupling from an intermediate‐centric perspective. We first summarise carbon‐side regulation approaches that control the generation, configuration, and availability of coupling‐active carbon intermediates. We then discuss nitrogen‐side regulation strategies that retain key nitrogen species and suppress their over‐hydrogenation toward ammonia. By organizing diverse catalyst design methods through the perspective of intermediate behavior, this chapter provides a unified framework for understanding how coordinated regulation of carbon and nitrogen reaction manifolds enables efficient urea electrosynthesis.

### 3.1 Regulation of Carbon‐Derived Intermediates

As for carbon‐containing species, surface‐bound *CO_2_ and *CO are widely regarded as the two most relevant carbon intermediates involved in C–N coupling during electrocatalytic urea synthesis. Notably, the optimal strategy for regulating carbon‐side activation is not universal but strongly depends on the specific C–N coupling pathway involved.

#### 3.1.1 Adsorption Configuration Regulation

**FIGURE 4:**
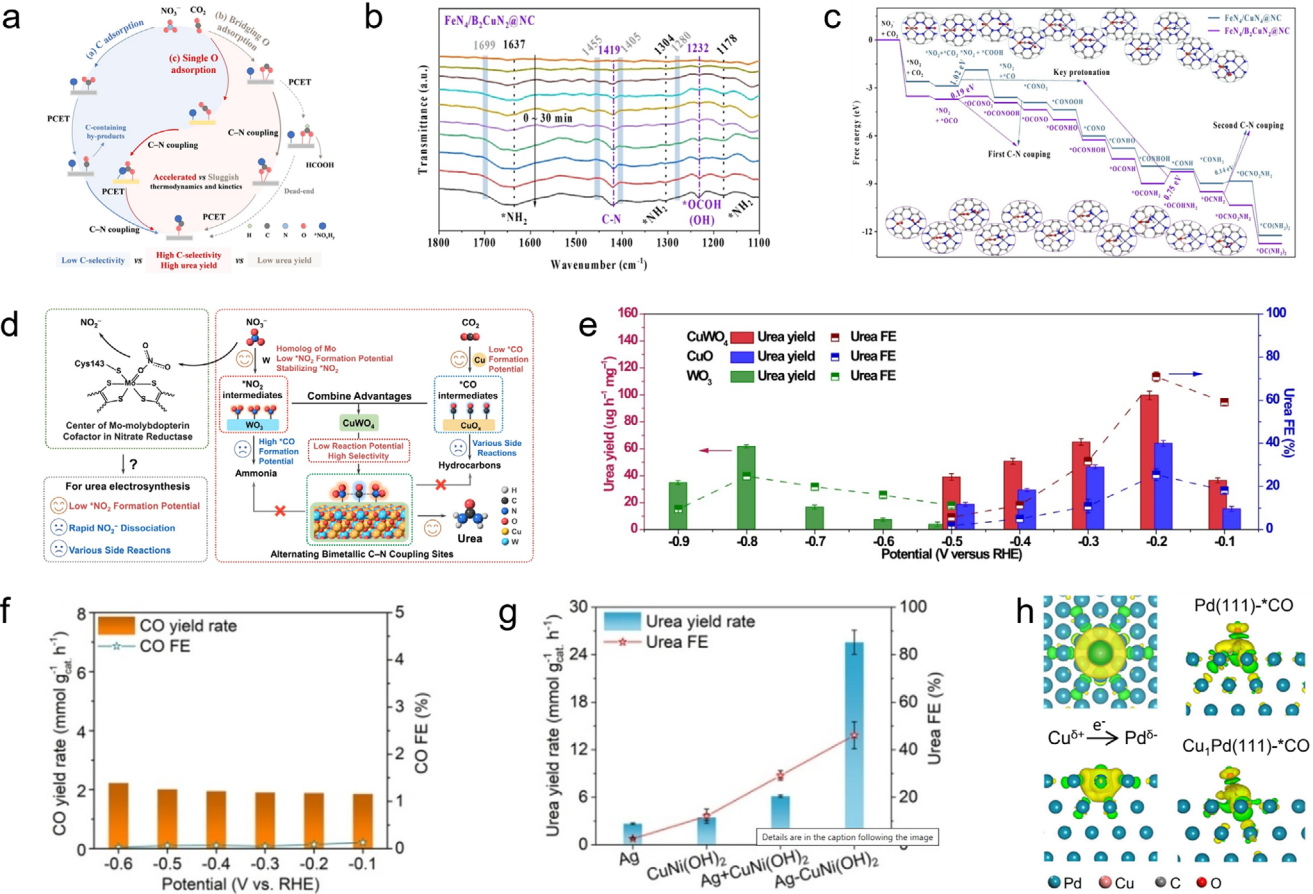
(a) The scheme of the single oxygen adsorption pathway for C–N coupling. (Adapted with permission from Ref. [54], copyright 2025 Wiley‐VCH). (b) In situ ATR‐FTIR spectra for FeN_4_/B_2_CuN_2_@NC. (Adapted with permission from Ref. [54], copyright 2025 Wiley‐VCH). (c) Free‐energy profiles of the CO_2_/NO_3_^−^ co‐reduction to urea on FeN_4_/CuN_4_@NC (blue) and FeN_4_/B_2_CuN_2_@NC (purple), together with the corresponding optimized reaction geometries. (Adapted with permission from Ref. [54], copyright 2025 Wiley‐VCH). (d) Bioinspired alternating Cu–W bimetallic site design in CuWO_4_ for urea electrosynthesis. (Adapted with permission from Ref. [33], copyright 2023 Springer Nature). (e) Faradaic efficiency and yield rates of urea for CuWO_4_ at different potentials. (Adapted with permission from Ref. [33], copyright 2023 Springer Nature). (f) CO yield rate and Faradaic efficiency of CuNi(OH)_2_ under CO_2_RR. (Adapted with permission from Ref. [72], copyright 2024 Wiley‐VCH). (g) Urea yield rates and Faradaic efficiencies obtained on Ag NPs, CuNi(OH)_2_ NSs, their physical mixture, and the Ag–CuNi(OH)_2_ composite. (Adapted with permission from Ref. [72], copyright 2024 Wiley‐VCH). (h) Differential charge density of Cu_1_Pd(111). (Adapted with permission from Ref. [33], copyright 2023 Springer Nature).

For the *CO_2_ + *NO_2_ → *CO_2_NO_2_ pathway, modulating the adsorption configuration of CO_2_ has been demonstrated to facilitate the C–N coupling process, as certain adsorption geometries are more favorable for effective bond formation with *NO_2_ [53, 54]. Zhou et al. reported a *CO_2_‐side activation strategy based on deliberate regulation of CO_2_ adsorption geometry (Figure 4a) [54]. By constructing dual‐metal hetero‐single‐atom sites with a FeN_4_/B_2_CuN_2_ coordination environment, the authors induced a strong antibonding interaction between CO_2_ and the Cu–B moiety, which selectively stabilizes a single O‐monodentate (*OCO) adsorption mode rather than conventional C‐linked or bridging O,O‐linked configurations.

Operando ATR‐FTIR spectroscopy, combined with isotope labelling experiments, revealed that this adsorption geometry enables the direct coupling between *OCO and *NO_2_ to form the *OCONO_2_ intermediate, effectively suppressing competing *CO_2_ and *COOH formation. Firstly, they employed liquid chromatography‐mass spectrometry/mass spectrometry (LC–MS/MS) to detect urea using ^13^CO_2_ or ^12^CO. They found that only m/z = 62 (^13^CO(NH_2_)_2_) was observed for FeN_4_/B_2_CuN_2_@NC, certifying that CO_2_ is the C‐containing coupling source rather than CO. Moreover, in situ ATR‐FTIR showed a peak at 1232 cm^−1^ for FeN4/B2CuN_2_@NC, which was assigned to *OCOH, and no *CO_2_ nor *COOH peaks were detected (Figure 4b), indicating the *OCO adsorption mode. KNO_2_, NH_2_OH, HCONH_2_, NH_3_, and NH_4_Cl were substituted for KNO_3_ to identify the nitrogen‐containing intermediates involved in the C–N coupling process. Only when KNO_2_ was used as a nitrogen source could urea be observed, which illustrates that *NO_2_ is the intermediate involved in C–N coupling. The results demonstrated that the C–N coupling mechanism on FeN_4_/B_2_CuN_2_@NC is *OCO + *NO_2_ → *CO_2_NO_2_. DFT calculations showed that the energy barrier of the first C–N coupling step *OCO and *NO_2_ was 0.19 eV, while that of *CO_2_ and *NO_2_ coupling was 1.02 eV (Figure 4c). As a result, compared with catalyst FeN_4_/CuN_4_@NC, which relies on *CO_2_/CO and *NO_2_ coupling, catalyst FeN_4_/B_2_CuN_2_@NC exhibited a higher Faradaic efficiency and urea yield, reaching 71.9% and 2072.5 µg h^−1^ mg_cat._^−1^, respectively. These comprehensive experimental and theoretical results collectively validate that the *OCO + *NO_2_ pathway, enabled by single‐O adsorption, is energetically and kinetically more favorable for C–N coupling. Similarly, Li et al. reported that CO_2_ activation occurs via the formation of a specific In–O–C–O–In adsorption configuration on exposed (100) facets, which facilitates electron transfer to the surface‐bound CO_2_ intermediate [53]. This coordination mode provides a favorable structural environment for direct C–N coupling between *NO_2_ and *CO_2_ intermediates, thereby promoting efficient urea formation.

The underlying reasons for promoting C–N coupling by regulating the adsorption configuration are as follows: the first is a difference in steric hindrance. When the adsorption configuration is the C‐linked *CO_2_, the carbon atom directly adsorbs on the catalyst surface and is spatially confined, leading to significant steric hindrance to coupling with nitrogenous intermediates. In contrast, in the O‐linked *OCO configuration, the carbon atom protrudes outward and is highly exposed, significantly reducing the steric hindrance and providing highly favorable geometric conditions for C–N coupling. The second reason may be that the C‐linked adsorption configuration allows direct electron transfer from the catalyst surface to the carbon atom, increasing its local electron density and reducing its electrophilicity. In contrast, in the O‐linked *OCO configuration, influenced by the electron‐withdrawing inductive effect of the metal‐oxygen interaction, the electron cloud shifts toward the oxygen atom [70]. This enhances the electrophilicity of the carbon atom, thereby thermodynamically promoting C–N coupling with the nucleophilic nitrogen intermediate.

#### 3.1.2 Promoting the Generation of *CO Intermediates

In addition to *CO_2_, *CO has also been identified as a viable carbon intermediate capable of undergoing coupling with nitrogen‐derived species during electrocatalytic urea synthesis [36, 71]. Therefore, by promoting the reduction of CO_2_ to produce *CO, the possibility of the coupling reaction between *CO and nitrogen intermediates can be effectively enhanced [10]. This strategy has been exemplified in several catalyst systems. For instance, CuWO_4_ has been reported to enhance the C–N coupling process by promoting the generation of *CO [33]. In this system, Cu sites are responsible for facilitating CO_2_ to *CO conversion, while adjacent W‐based oxide motifs preferentially stabilize nitrogen‐derived intermediates such as *NO_2_ (Figure 4d). Researchers compared the urea production behaviors of WO_3_ and CuWO_4_ catalysts and found that the urea yield on WO_3_ reached its maximum at −0.8 V vs. RHE (61.7 ± 1.2 µg h^−1^ mg^−1^). However, after introducing Cu sites that could promote the generation of *CO, the peak urea yield of CuWO_4_ (99.5 ± 3.0 µg h^−1^ mg^−1^) significantly shifted to −0.2 V vs. RHE, and it was significantly higher than that of WO_3_ (Figure 4e). This result indicates that by enhancing the generation of *CO, the C–N coupling can be promoted.

Ye et al. achieved a significant improvement in urea selectivity by introducing Ag sites that could promote the generation of *CO into the CuNi(OH)_2_ system [72]. The CO_2_ reduction test on CuNi(OH)_2_ only yields trace amounts of CO, indicating a low CO_2_ reduction activity (Figure 4f). Consequently, CuNi(OH)_2_ exhibited a lower urea yield in the co‐reduction system. In contrast, after loading Ag nanoparticles with CO_2_ reduction activity to *CO on CuNi(OH)_2_, Ag‐CuNi(OH)_2_ showed a significantly increased urea yield (Figure 4g). This result further highlights the significant role of the *CO generation ability in regulating the C–N coupling process.

In the system where *CO serves as the primary coupling intermediate, promoting the reduction of CO_2_ to *CO is highly beneficial for increasing the surface *CO coverage. From a kinetic perspective, the rate of C–N coupling is typically positively correlated with the *CO coverage.

#### 3.1.3 Regulation of *CO Adsorption Strength

Beyond improvements in *CO generation, regulating the adsorption strength of *CO on catalyst surfaces is another important strategy for enhancing C–N coupling [68]. Specifically, overly weak *CO adsorption results in rapid desorption, while excessively strong binding favors further hydrogenation to hydrocarbons or oxygenates, thereby limiting the participation of carbon species in urea formation [24, 39, 61, 73]. Therefore, an optimal *CO adsorption strength is required to balance surface stabilization and chemical reactivity. For instance, Zhao et al. effectively modulated the adsorption intensity of the *CO intermediate by regulating the exposed crystal planes of the Cu_2_O catalyst [73]. DFT calculations indicated that the binding of *CO to the Cu(110) surface was weak, leading to the desorption of *CO and weakening the effective coupling between *CO and *NH species. On the contrary, on the Cu(100) surface, the C–N coupling process is more likely to occur. This is mainly attributed to the fact that the Cu(100) surface has a stronger adsorption ability for *CO, thereby providing favorable conditions for *CO to participate in the subsequent C–N bond formation.

The strategy of promoting C–N coupling by regulating the adsorption strength of *CO was also demonstrated in the work of Xu et al. [63]. On the Pd_4_Cu_1_/FeNi(OH)_2_ catalyst, the Pd sites serve to stabilize the *CO intermediate and prevent its premature desorption, thus providing more favorable reaction conditions for C–N coupling between *CO and the nitrogen source intermediate. DFT calculations revealed that on the Cu_1_Pd(111) single‐atom alloy surface, the free energy change for *CO desorption is increased relative to pure Pd surfaces, indicating that *CO is more strongly retained at the catalyst surface. Differential charge density analysis on the Cu_1_Pd(111) facet (Figure 4h) shows a clear electron delocalization from the Cu atom to the surrounding Pd atoms, which can be attributed to the higher electronegativity of Pd. This trend is further supported by Bader charge analysis, indicating that the Cu atom transfers approximately 0.21 e^−^ to neighboring Pd atoms on the Cu_1_Pd(111) facet, while the pristine Pd(111) facet maintains an almost neutral charge distribution. Therefore, electrophilic *CO preferentially stabilizes on the electron‐rich Pd sites. As a result of the enhanced *CO adsorption capacity, the C–N coupling efficiency at the Pd_4_Cu_1_/FeNi(OH)_2_ site significantly increased, achieving a urea Faraday efficiency of up to 76.2% at −0.5 V vs. RHE. Additionally, a very small amount of CH_4_ was detected within the potential range of −0.1 to −0.3 V, which is also related to the enhanced *CO adsorption strength.

#### 3.1.4 Spatial and Interfacial Regulation of *CO for C–N Coupling

Even when *CO is sufficiently generated and stabilized, effective C–N coupling critically depends on whether *CO is spatially positioned in close proximity to nitrogen‐derived intermediates. In conventional dual‐site catalysts, the diffusion length of surface‐bound *CO is typically very short, and *CO migration across heterogeneous interfaces is energetically unfavorable [74]. As a result, *CO and nitrogen‐derived intermediates remain spatially separated, which severely limits effective C–N coupling and hampers urea formation [75]. Therefore, engineering spatial and interfacial environments that confine or localise *CO near coupling‐active nitrogen sites represents another promising strategy for promoting C–N coupling toward urea synthesis [76, 77].

**FIGURE 5:**
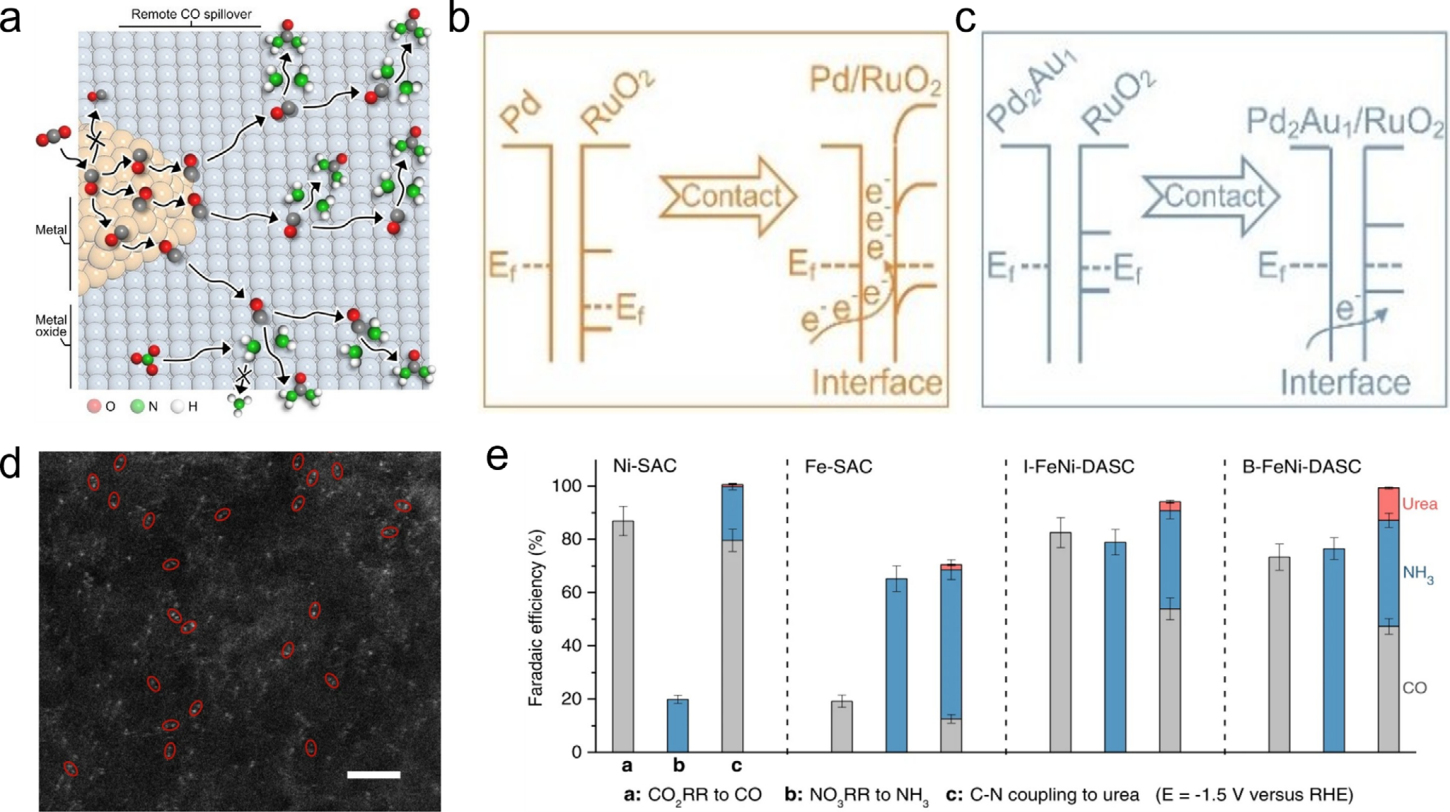
(a) Schematic illustration of remote *CO spillover‐enhanced tandem urea electrosynthesis on metal/metal oxide catalysts. (Adapted with permission from [77], copyright 2025 Wiley‐VCH). Interfacial electronic configuration analysis of (b) Pd/RuO_2_ (Δ*Φ* = 0.44 eV) and (c) Pd_2_Au_1_/RuO_2_ (Δ*Φ* = 0.05 eV). (Adapted with permission from [77], copyright 2025 Wiley‐VCH). (d) High‐Angle Annular Dark‐Field Scanning Transmission Electron Microscopy (HAADF‐STEM) image of B‐FeNi‐DASC. (Adapted with permission from [76], copyright 2022 Springer Nature). (e) The product distributions of CO_2_RR, NO_3_RR and urea synthesis on Ni‐SAC, Fe‐SAC, I‐FeNi‐DASC, and B‐FeNi‐DASC at −1.4 V vs. RHE. (Adapted with permission from [76], copyright 2022 Springer Nature).

To overcome the spatial separation between *CO produced at CO_2_RR‐active sites and nitrogen‐derived intermediates, Li et al. introduced a *CO spillover strategy that enables effective migration of *CO toward coupling‐active sites, thereby facilitating urea synthesis (Figure 5a) [77]. In this system, palladium–gold alloys (Pd_2_Au_1_) and ruthenium dioxide (RuO_2_) serve as CO_2_RR‐active and NO_3_RR‐active components, respectively, enabling efficient generation of *CO and *NH_2_ intermediates. The Pd_2_Au_1_/RuO_2_ interface exhibits a relatively lower work function than Pd/RuO_2_ (Figure 5b,c). The decreased work function suppresses excessive interfacial charge accumulation, resulting in a lower energetic barrier for *CO migration. Accordingly, *CO generated on Pd_2_Au_1_ sites can more readily spillover across the interface toward RuO_2_, where nitrogen‐derived intermediates are preferentially stabilized. This facilitated *CO spillover enables effective spatial coupling between carbon‐ and nitrogen‐derived intermediates, ultimately promoting urea formation.

Different from the aforementioned *CO spillover approach, Zhao et al. achieved the coupling of CO_2_RR and NO_3_RR functionalities by constructing a Ni–Fe diatomic pair (B‐FeNi‐DASC), in which the carbon‐ and nitrogen‐reduction sites are intrinsically linked at the atomic scale (Figure 5d) [76]. This design integrates the CO_2_RR and NO_3_RR active sites and deliberately regulates their spatial proximity, allowing the coupling of the C intermediate and the N intermediate. Specifically, Ni–SACs exhibit high activity toward the reduction of CO_2_ to CO, while Fe–SACs show favorable performance in converting NO_3_^−^ to NH_3_. However, when these two catalytic sites exist independently, the catalytic ability is inefficient in promoting the necessary C–N coupling for the formation of urea. Although the isolated diatomic Fe–Ni electrocatalyst (I‐FeNi‐DASC) is active for CO_2_RR and NO_3_RR individually, its contribution to urea electrosynthesis remains minimal. In contrast, the B‐FeNi‐DASC demonstrates markedly improved performance in urea electrosynthesis, indicating that Fe–Ni pairs play a vital role in enhancing the reaction kinetics of the C–N coupling process and overall reaction efficiency (Figure 5e).

### 3.2 Regulation of Nitrogen‐Containing Intermediates

Carbon‐side regulation focuses on the generation, stabilization, and spatial management of coupling‐active carbon intermediates; efficient urea electrosynthesis also critically depends on the behavior of nitrogen‐derived species [78, 79]. As discussed in Section 2, several nitrogen intermediates, including *NO_2_, *NO, *NH_2_, have been proposed to participate in C–N coupling through different reaction pathways. In practice, these intermediates tend to desorb readily or undergo rapid hydrogenation, which limits their effective interaction with carbon intermediates [80]. Accordingly, design strategies are required to address the instability of nitrogen intermediates during urea electrosynthesis. Generally, the intermediate can be prevented from desorbing or from being further reduced to NH_3_ by regulating its adsorption strength and inhibiting excessive hydrogenation. In the following sections, we have summarised several representative design strategies.

#### 3.2.1 Adsorption Strength Regulation of Nitrogen Intermediates

Enhancing the surface retention of coupling‐active nitrogen intermediates, such as *NO_2_, is an effective nitrogen‐side regulation strategy for enabling efficient C–N coupling. During electrochemical reduction, *NO_2_ tends to desorb from catalyst surfaces [81, 82], resulting in an insufficient population available for subsequent C─N bond formation. Insufficient stabilization of *NO_2_ often limits its effective coupling with carbon species.

**FIGURE 6:**
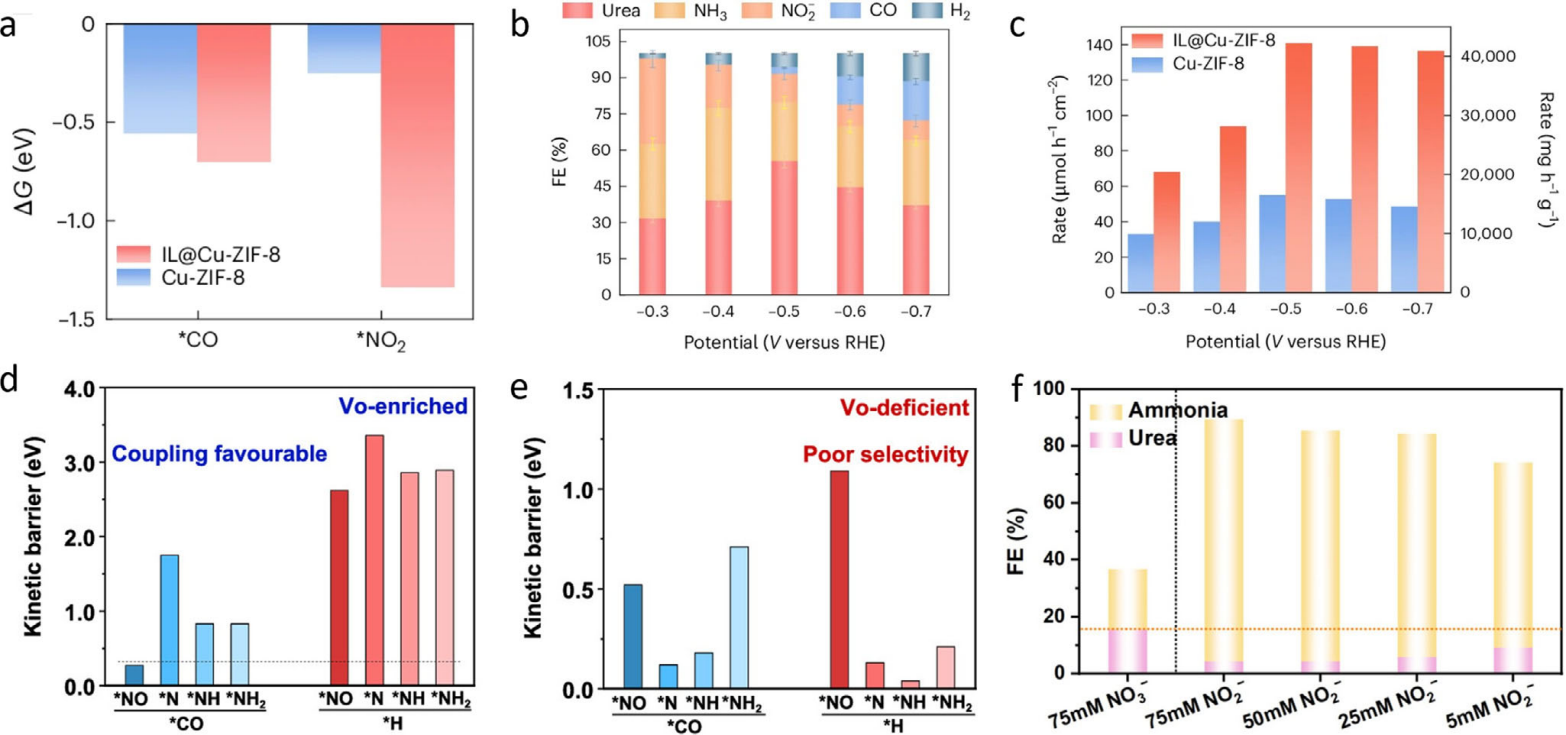
(a) The adsorption energies of *CO and *NO_2_ on IL@Cu‐ZIF‐8 and Cu‐ZIF‐8. (Adapted with permission from Ref. [57], copyright 2026 Springer Nature). (b) Faradaic efficiency of product distribution with applied potential for IL@Cu‐ZIF‐8. (Adapted with permission from Ref. [57], copyright 2026 Springer Nature). (c) Urea production rate with applied potential. (Adapted with permission from Ref. [57], copyright 2026 Springer Nature). Free energy profiles comparing the coupling of *NO, *N, *NH, and *NH_2_ with *CO, and the corresponding protonation steps, on V_O_‐enriched (d) and V_O_‐deficient CeO_2_ (e). (Adapted with permission from Ref. [38], copyright 2022 American Chemical Society). (f) Faradaic efficiencies of urea and NH_4_^+^ during CO_2_ and NO_3_^−^/NO_2_^−^ co‐reduction at different reactant concentrations. (Adapted with permission from Ref. [66], copyright 2025 American Chemical Society).

Adsorption strength regulation focuses on tuning the interaction between nitrogen intermediates and catalytic surfaces to suppress their premature loss and maintain sufficient surface availability for coupling with carbon species. Recent studies have confirmed that this strategy effectively promotes C–N coupling [56, 57]. For instance, Yin et al. enhanced the adsorption capacity of *CO and *NO_2_ by modifying Cu‐ZIF‐8 with ionic liquids [57]. Compared with the original Cu‐ZIF‐8 (with *CO: −0.56 eV, *NO_2_: −0.25 eV), the IL@Cu‐ZIF‐8 modified by ionic liquids exhibits stronger adsorption capabilities for *CO and *NO_2_, with its adsorption energies reduced to −0.70 eV and −1.34 eV, respectively (Figure 6a). This enhanced adsorption stabilizes reactive intermediates and further facilitates subsequent C–N coupling. As a result, IL@Cu‐ZIF‐8 achieves a urea yield up to 140 µmol h^−1^ cm^−2^ (∼42 000 mg h^−1^ g_cat_^−1^) with a Faradaic efficiency of 55.3% at −0.5 V vs. RHE (Figure 6b,c). Moreover, Zhang et al. demonstrated that Bi‐doped Cu catalyst exhibits enhanced *NO_2_ adsorption relative to pristine Cu, which increases the surface population of *NO_2_ and consequently improves urea selectivity [56]. These results collectively demonstrate that reinforcing *NO_2_ adsorption is an effective nitrogen‐side strategy to prevent premature intermediate loss and promote C–N coupling.

#### 3.2.2 Suppression of Over‐Hydrogenation of Nitrogen Intermediates

In addition to adsorption regulation, efficient urea electrosynthesis requires suppressing the over‐hydrogenation of nitrogen intermediates during nitrate reduction, as excessive hydrogenation readily drives these species toward ammonia formation at the expense of C–N coupling [83]. Once adsorbed, nitrogen intermediates such as *NO_2_, *NO, and *NH_2_ can readily undergo sequential proton‐coupled electron‐transfer steps, thermodynamically favoring deep hydrogenation to NH_3_ under reductive conditions. This competitive pathway substantially consumes nitrogen flux and reduces the availability of coupling‐active intermediates for C−N bond formation.

Therefore, it is necessary to regulate the reaction process reasonably to inhibit the excessive hydrogenation of nitrogen intermediates, thereby preventing their deep reduction to NH_3_. Previous studies have shown that limiting the continuous hydrogenation of nitrogen intermediates is essential for preventing their diversion toward downstream intermediates and preserving their involvement in C–N coupling pathways [84]. Wei et al. suppress the further hydrogenation of N‐containing intermediates through the oxygen vacancies (V_O_) in CeO_2_, thereby promoting its coupling with *CO rather than hydrogenation toward downstream intermediates [38]. For vacancy‐deficient CeO_2_, the calculated energy barrier for *N–*CO coupling is lowest in all possible C–N coupling steps. However, *N is more prone to hydrogenation, which redirects the reaction toward downstream intermediates and ultimately leads to low urea selectivity (Figure 6d). After the introduction of V_O_, the activation energy barrier for the hydrogenation of nitrogen intermediates significantly increases. At this point, the coupling of *NO and *CO becomes the most likely C–N coupling step, and its reaction energy barrier (0.27 eV) is lower than that of the hydrogenation process of *NO (Figure 6e). In addition, to verify the promotion of suppressing the hydrogenation process on the formation of urea through C–N coupling, Liu et al. conducted comparative experiments on the FeP_0.9_S_2.9‐x_ catalyst using NO_3_^−^ and NO_2_^−^ as nitrogen sources. The results showed that when NO_2_^−^, which is more prone to the hydrogenation reaction, was used as the nitrogen source, the Faradaic efficiency of urea decreased significantly, while the selectivity of ammonia increased markedly (Figure 6f) [66]. DFT calculations further showed that the free‐energy barriers for *NH_2_ hydrogenation and *NH_2_–CO coupling are comparable, indicating that suppressing *NH_2_ over‐hydrogenation is essential for maintaining an effective C–N coupling pathway. Collectively, these studies demonstrate that suppressing the over‐hydrogenation of nitrogen intermediates is a general nitrogen‐side regulation strategy for preserving coupling‐active species and promoting efficient C–N bond formation during urea electrosynthesis.

In this section, regulation strategies for electrocatalytic urea synthesis were summarised from both the carbon and nitrogen perspectives. Carbon‐side regulation primarily aims to control the generation, stabilization, and availability of coupling‐active carbon intermediates, whereas nitrogen‐side regulation focuses on retaining key nitrogen intermediates and suppressing their over‐hydrogenation toward ammonia. While these strategies are discussed separately for clarity, increasing evidence suggests that high urea selectivity often arises from the simultaneous implementation of multiple regulation directions, in which coordinated control over intermediate generation, retention, and reaction pathways across both manifolds is achieved.

## 4 In Situ Characterization Techniques Uncovering C–N Coupling Mechanisms

**FIGURE 7:**
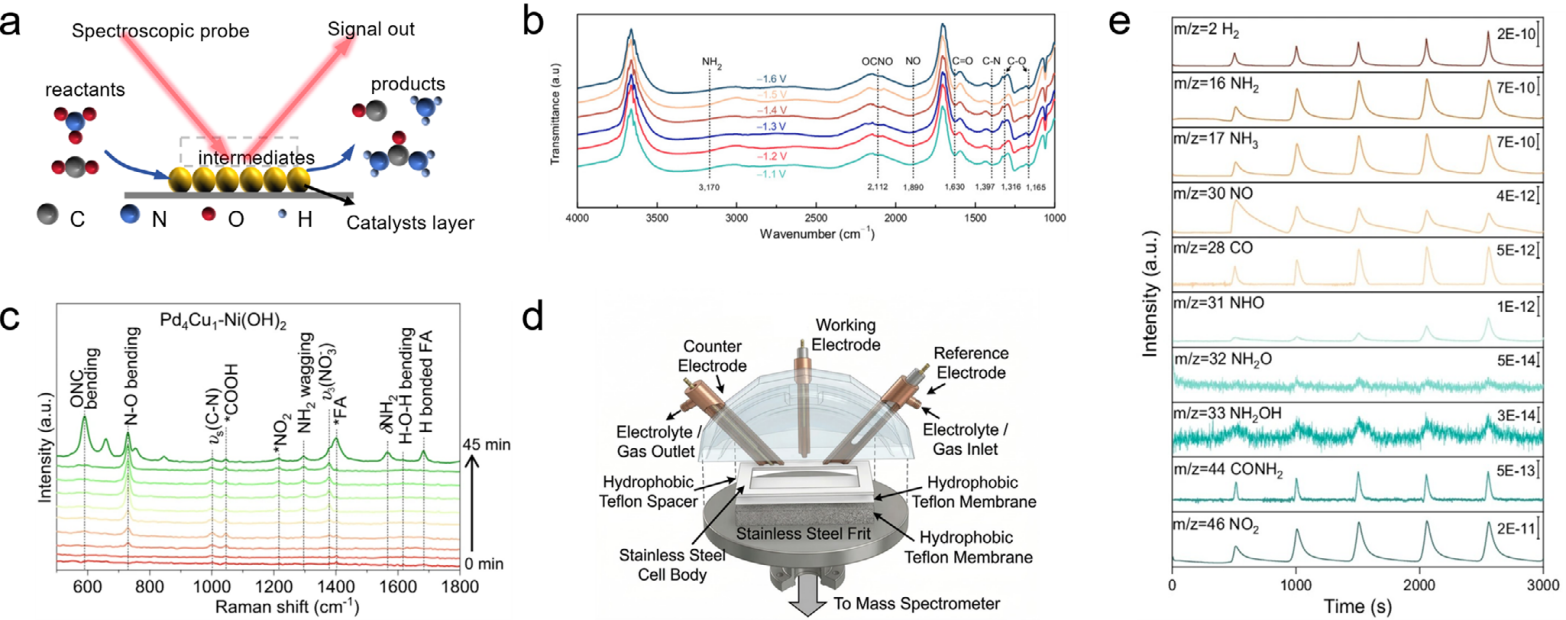
(a) Schematic illustration of in situ characterization for probing reaction intermediates. (b) In situ infrared spectroscopy of m‐Cu_2_O. (Adapted with permission from Ref. [87], copyright 2023 Chinese Chemical Society). (c) In situ Raman spectroscopy of Pd_4_Cu_1_–Ni(OH)_2_. (Adapted with permission from Ref. [63], copyright 2023 Springer Nature). (d) Schematic illustration of the DEMS setup. (f) Online DEMS analysis of CuSn/CS−1. (Adapted with permission from Ref. [79], copyright 2025 Springer Nature).

We have summarised a variety of C–N coupling pathways and intermediate regulation strategies that have been proposed. Most mechanistic insights into electrocatalytic urea synthesis are inferred from in/ex situ analysis and theoretical calculations [85]. Therefore, in situ characterization is essential for elucidating the detailed reaction mechanism. Techniques such as in situ Fourier‐transform infrared spectroscopy, Raman spectroscopy, and online DEMS enable real‐time monitoring of reaction intermediates [46], providing critical insights into the dynamic catalytic process and C–N coupling mechanism (Figure 7a). In this section, we introduce these characterization techniques and summarise representative studies in which they have been applied to probe C–N coupling mechanisms.

### 4.1 In Situ Fourier‐Transform Infrared Spectroscopy

In situ FTIR spectroscopy is a widely used technique in electrocatalysis, which enables real‐time monitoring of surface‐absorbed intermediates under reaction conditions [86]. During the co‐reduction of CO_2_ and NO_3_^−^, in situ FTIR spectroscopy has been employed to detect C‐containing, N‐containing, and C–N coupling intermediates. By monitoring intermediates along with their appearance, disappearance, intensity enhancement, and spectral shifts, in situ FTIR provides supportive evidence for elucidating the reaction pathway. For example, Qiu et al. detected two peaks at ∼3170 and ∼1890 cm^−1^ on multihole‐Cu_2_O (m‐Cu_2_O) through in situ FTIR spectra, which were assigned to the stretching vibrations of *NH_2_ and *NO, respectively (Figure 7b) [87]. Additionally, peaks at ∼1630 cm^−1^ and in the 1165–1316 cm^−1^ range corresponded to the stretching modes of C═O and C−O, respectively, indicating the presence of carbonaceous intermediates. Notably, the peak at approximately 2112 cm^−1^ is attributed to the vibrational mode of *ONCO that is likely formed through the coupling of *CO and *NO. These results demonstrate that m‐Cu_2_O is capable of simultaneously activating and reducing CO_2_ and NO_3_^−^, while also facilitating the coupling between *CO and *NO intermediates.

### 4.2 In Situ Raman Spectroscopy

In situ Raman spectroscopy is an effective technique for probing both the structural evolution of catalysts and the formation of reaction intermediates during electrocatalytic processes [88]. In situ Raman spectroscopy enables the investigation of catalyst structural changes under different applied potentials or over time at a fixed potential, as well as the adsorption behavior of reaction intermediates on the catalyst surface [89]. In the field of electrocatalytic CO_2_ and NO_3_^−^ co‐reduction, in situ Raman spectroscopy has been rarely employed to monitor catalyst reconstruction during the reaction. Therefore, this section focuses exclusively on the application of in situ Raman spectroscopy for identifying surface‐bound intermediates and uncovering mechanistic insights into the C–N coupling process. Using in situ Raman spectroscopy at −0.5 V vs. RHE from 0 to 45 min, Ye et al., confirmed the C–N coupling mechanism on Pd_4_Cu_1_‐Ni(OH)_2_ catalyst [63]. The appearance of two vibrational peaks at 1216 and 1296 cm^−1^ can be attributed to the wagging modes of *NO_2_ and *NH_2_ intermediates, respectively. In addition, the peaks at 590, 1597, and 1683 cm^−1^ that appeared with high intensity at 45 min are attributed to the OCN bending mode, the δNH_2_ vibration of formamide (HCONH_2_), and the H‐bonded formamide signal, respectively (Figure 7c). Combined with the characteristic peak around 1000 cm^−1^, which is attributed to the C─N bond in urea, it can be inferred that *CONH_2_ serves as a key intermediate in the urea formation pathway. Additionally, Zhao et al. employed in situ Raman spectroscopy to understand the C–N coupling mechanism on the CuWO_3_ catalyst [33]. Two characteristic vibrational peaks at 1428 and 1979 cm^−1^ were observed, which were assigned to surface‐adsorbed *NO_2_ and *CO intermediates, respectively. The intensities of these peaks reached their maximum at −0.2 V vs. RHE, indicating a high coverage of both species. The maximum urea Faradaic efficiency is observed at this potential, implying that the simultaneous high coverage of *NO_2_ and *CO facilitates their coupling and promotes C─N bond formation. These results underscore the value of in situ Raman spectroscopy for elucidating the C–N coupling mechanism during electrochemical urea synthesis. However, the practical application of in situ Raman spectroscopy in probing surface intermediates remains limited due to the typically low surface coverage of key intermediates and their intrinsically weak Raman activity.

### 4.3 Online Differential Electrochemical Mass Spectrometry

Differential electrochemical mass spectrometry (DEMS) is a powerful in situ analytical technique that enables the real‐time detection and quantification of volatile or gaseous intermediates and products generated during electrochemical reactions. By coupling an electrochemical cell directly to a mass spectrometer via a gas‐permeable membrane, DEMS enables continuous monitoring of reaction species under working conditions without disturbing the reaction environment (Figure 7d). This technique has been extensively employed in CO_2_ reduction and NO_3_^−^ reduction to elucidate reaction pathways by identifying key intermediates and products such as *CO, *HCO, *NO_2_, *NO, *NH_2_OH, and *NH_3_. However, intermediates formed during C–N coupling are often difficult to detect due to their low volatility, short lifetimes, and low coverage. Therefore, while DEMS provides valuable insights into individual half‐reactions and precursor species, it is typically used as a supplemental tool to probe C–N coupling mechanisms. For instance, Wu et al., proposed a C–N coupling mechanism on CuSn/CS−1 [79]. Through online DEMS analysis, the CONH_2_ signal was observed under applied potential, with no CONO_2_, CONH, or CONHO signals. The results suggest that *CO and *NH_2_ are dominant intermediates in the C–N coupling process (Figure 7e). However, because many crucial reaction intermediates cannot be detected by DEMS, it remains an auxiliary technology to elucidate the C–N coupling mechanism.

## 5 Conclusion and Perspectives

Coupling CO_2_ and NO_3_^−^ electroreduction to synthesize urea offers a sustainable route toward carbon neutrality and efficient nitrogen removal from wastewater. This approach offers a promising strategy for producing green chemicals and promoting environmental remediation. This review focuses on recent advances in the electrocatalytic co‐reduction of CO_2_ and NO_3_^−^, emphasizing C–N coupling mechanisms, the regulation of reaction intermediates, and in situ characterization approaches. Firstly, five representative C–N coupling mechanisms were reviewed, including: (i) the reaction between *CO_2_ and *NO_2_ forming *CO_2_NO_2_, (ii) *CO with *NO_2_ forming *CONO_2_, (iii) *CO with *NO yielding *OCNO, (iv) *CO coupling with *NH_2_ to form *CONH_2_, and (v) direct coupling between gaseous CO_2_ and N intermediates. In addition, several intermediate‐regulation strategies that promote C–N coupling were discussed. Finally, in situ electrochemical characterization techniques employed to elucidate the C–N coupling mechanisms were summarized. However, the current understanding of the C–N coupling mechanism is still limited, mainly because of the limitations of current characterization methods and theoretical calculation accuracy. In this regard, the following potential perspectives may contribute to a deeper understanding of the C–N coupling mechanism, guide rational catalyst design and ultimately enhance urea production performance.

### 5.1 Developing More Advanced in Situ/Operando Characterization Techniques

Currently, in situ characterization methods cannot directly elucidate the C–N coupling mechanism [90]. Understanding this process relies predominantly on detected intermediate signals, integrated with theoretical calculations and relevant experimental observations for indirect interpretation. This limitation is mainly because the existing in situ characterization techniques are unable to directly observe the initial steps of the formation of the C─N bond. For example, the observation of *CONH_2_ in in situ FTIR does not definitively prove that the C–N coupling occurred between *CO and *NH_2_, as the coupling might have taken place at an earlier stage, and *CONH_2_ could merely be a downstream intermediate formed after subsequent hydrogenation steps. To better understand the C–N coupling mechanism, it is necessary to develop characterization techniques that can more effectively monitor intermediates during the co‐reduction of CO_2_ and NO_3_^−^.

Improving temporal resolution and enhancing signal sensitivity are two promising development directions for existing in situ characterizations. Under electrochemical operating conditions, many C–N coupling intermediates are highly transient and have low surface coverage, making them difficult to detect by conventional steady‐state in situ spectroscopy [91]. Accordingly, improving temporal resolution through the integration of time‐resolved or rapid‐scan spectroscopic techniques therefore represents a critical step toward capturing these short‐lived intermediates before they evolve into more stable products. Meanwhile, strategies that enhance spectroscopic sensitivity are required to access intermediates with intrinsically weak signals. In this regard, enhanced Raman surface‐enhancement approaches provide a representative example. The amplified vibrational signals of adsorbed reaction intermediates enable the detection of previously inaccessible species, particularly those formed at the early stages of C–N coupling. In addition to the improvements to the existing characterizations, the development of new in situ and operando characterizations is equally important. Several emerging approaches, for example, in situ soft X‐ray spectroscopy and electrochemical scanning probe microscopies, offer new opportunities for probing C–N coupling processes. By resolving electronic structure, surface bonding, and reaction kinetics, these advanced techniques may ultimately enable direct observation of C─N bond formation.

Furthermore, the inability to directly observe the C–N coupling process might also be due to the fact that in situ characterizations cannot fully reflect the actual operating conditions. Although many in situ characterizations are conducted in simulated reaction environments, they differ from the electrode polarisation state and local environmental conditions (local pH and ion concentration) in the actual reaction process. The intermediate signal obtained under non‐realistic conditions may not correspond to the key intermediates in the actual reaction pathway. Therefore, even if certain C–N coupling species are detected under in situ conditions, they may not be the C–N intermediates in the actual process. In contrast, *operando* characterizations can better reflect the information of intermediates under actual reaction conditions. Therefore, developing *operando* characterization techniques applicable to the electrochemical co‐reduction of CO_2_ and NO_3_^−^/NO_2_^−^ to urea is expected to help better explore the C–N coupling mechanism.

The complex solid–liquid interface environment often leads to severe background signal interference and peak overlap in in situ/operando spectra. Therefore, in situ/operando characterization techniques should be combined with isotope labelling experiments (^13^CO_2_, ^15^NO_3_^−^). By comparing and observing the isotope shifts of characteristic peaks of key intermediate species, interference from interface solvent molecules and background ions would be effectively excluded. This avoids incorrect identification of the intermediate species and provides more accurate experimental evidence for the C–N coupling mechanism.

It is worth noting that because multiple C–N coupling pathways may coexist in this reaction system, even if all potential C–N coupling intermediates are observed (such as *CO_2_NO_2_, *COOHNO_2_, *CONO_2_), the species appearing earliest along the pathway cannot be considered as the key intermediate for the dominant C–N coupling. Therefore, establishing a quantitative relationship between spectral peak intensity and surface coverage is helpful for accurately analyzing the actual coverage of each intermediate, and thereby determining which C–N coupling mechanism is most dominant.

### 5.2 Improving the Reliability and Relevance of Theoretical Calculations

DFT calculations are the main method for studying the C–N coupling mechanisms. It can directly simulate the C–N coupling process and simulate the free energy for every hydrogenation and coupling step. However, current theoretical calculations still face some challenges in understanding the C–N coupling mechanism. Generally, the determination of reaction mechanisms relies on calculating the free energy of reaction pathways on ideal catalyst surfaces [92]. However, ideal models are difficult to accurately reflect realistic electrochemical conditions.

In order to enhance the accuracy of theoretical calculations in describing realistic reaction conditions, future efforts can be made to improve physical model construction and kinetics analysis. For the model construction, the theoretical calculations should gradually translate from idealized models to realistic electrochemical reaction environments. Introducing key interface factors, such as electric field effects, solvent structure, and surface structure flexibility during the calculation process, helps to enhance the accuracy of theoretical models in describing the actual reaction conditions.

In addition, the analysis should not only focus on adsorption energies and reaction energy barriers obtained from thermodynamic analyses. Instead, kinetic analysis should also be considered. For the C–N coupling reaction, some important intermediates have short lifetimes and low surface coverage. These species may be difficult to couple with other intermediates, although the energy barrier for their coupling is relatively low. So, thermodynamic analysis is often difficult to depict real reactions accurately. Therefore, to determine the actual C–N coupling steps, it is essential to calculate the C–N coupling mechanism through introducing kinetic analysis.

### 5.3 Synergistic of Computational Chemistry and Experiment for Mechanism Study

Studying the reaction process together with computational calculation and experimental characterizations is promising to further reveal the C–N coupling mechanism. In past decades, although in situ characterization techniques and theoretical calculations have developed rapidly, the integration between theory and experiment remains insufficient. Currently, synergy between theoretical calculations and experiment is still mainly as complementary approaches, rather than a fully integrated framework with reciprocal feedback and constraint.

To further integrate computational calculations and experiments, several key descriptors that can be tested experimentally and effectively distinguish different C–N coupling mechanisms should be employed. Such descriptors should enable computational calculations and experiments to constrain each other and enhance their cooperation in analyzing C–N coupling mechanisms. In this context, the physical model is established to generate concrete descriptors that can be directly evaluated based on experimental data, rather than only to identify thermodynamically favorable reaction pathways. Correspondingly, experiments should not only demonstrate the existence of intermediates but also quantify reaction trends and their responses to reaction conditions. By strengthening the connection between computational calculation and experiments, they can clearly and effectively uncover crucial mechanisms in C–N coupling, which further guides the design of urea electrosynthesis catalysts.

## Author Contributions

**Ziyun Wang**: writing – review and editing, conceptualization, writing – original draft, funding acquisition, resources, supervision, formal analysis, project administration. **Xingbao Chen**: writing – original draft, validation, visualization, writing – review and editing. **Zhuo Li**: writing – original draft, visualization. **Paul Kilmartin**: project administration, supervision, formal analysis, visualization, resources. **Lebin Cai**: visualization. **Bao Yu Xia**: writing – original draft, writing – review and editing, supervision.

## Conflicts of Interest

The authors declare no conflicts of interest.

## Data Availability

Data sharing not applicable to this article as no datasets were generated or analysed during the current study.
